# Central Giant Cell Granuloma of the Mandible: A Case Report

**DOI:** 10.7759/cureus.57729

**Published:** 2024-04-06

**Authors:** Ojas V Desai, Rajesh Kshirsagar, Vikram Singh, Vivek S Nair, Vikrant Sane, Saurabh Jain, Roshan Agarwal

**Affiliations:** 1 Oral and Maxillofacial Surgery, Bharati Vidyapeeth Dental College and Hospital, Pune, IND; 2 Dental Administrator, Forest Hill Dental, Ontario, CAN

**Keywords:** intralesional corticosteroid, surgical curettage, osteolytic lesion, giant cell reparative granuloma, central giant cell granuloma

## Abstract

This article presents a clinical case of a central giant cell granuloma (CGCG) resembling a periapical lesion of endodontic origin. A 39-year-old, otherwise healthy male patient was referred to the department of oral and maxillofacial surgery for its diagnosis and subsequent management. The patient presented with an asymptomatic, progressively increasing intraoral swelling associated with the mandibular left para-symphysis region. On radiographic evaluation, a unilocular radiolucent lesion involving 33-34 teeth was noted. An incisional biopsy presented a giant cell lesion, following which surgical curettage was done. Histopathological examination was in accordance with the diagnosis of CGCG. Therefore, it is imperative for clinicians to accurately diagnose and rule out similarly presenting lesions.

## Introduction

Central giant cell granuloma (CGCG) is an intraosseous, osteolytic lesion of the jaw, best classified as a benign neoplasm because of its unpredictable and sometimes aggressive behaviour [1,2]. It is defined by the WHO as an intraosseous lesion having a collection of thick connective tissues and cells that occasionally contain trabeculae of woven bone, several haemorrhagic foci, and an aggregation of multinucleated giant cells. The CGCG is an uncertain and idiopathic lesion; however, factors like local trauma, intraosseous haemorrhage, and genetic abnormalities are considered possible causes [3]. CGCG accounts for up to 7% of tumours present in the mandible and maxilla [4,5]. It occurs more frequently in females than in males [6].

Jaffe et al. in 1953 called this lesion “giant cell reparative granuloma [7]," but the word “reparative” has been dissolved as Chuong et al. were able to distinguish between aggressive and non-aggressive giant cell lesions [8,9]. Giant cell granuloma and its associated lesions in the jaws are grouped under a single category but with varied clinical behaviour consisting of both simple reactive to neoplasm and rarely manifesting as aggressive malignant lesions. The reactive secondary changes in the lesions give a false image of malignancy, which requires expertise to recognise the very nature of the lesion, therefore complicating the differential diagnosis with the presence of giant cells in unrelated bone lesions [1]. The most common treatment for small, non-aggressive CGCG of the jaws remains curettage; however, several therapies have been proposed in the literature, which includes intralesional injections of calcitonin, interferons, bisphosphonates, and corticosteroids [10] to more severe interventions such as excisional biopsies, surgical enucleation with curettage, or resection [11].

This article presents a clinical case of a CGCG resembling a periapical lesion of endodontic origin in the mandibular left para-symphysis region.

## Case presentation

**Figure 1:**
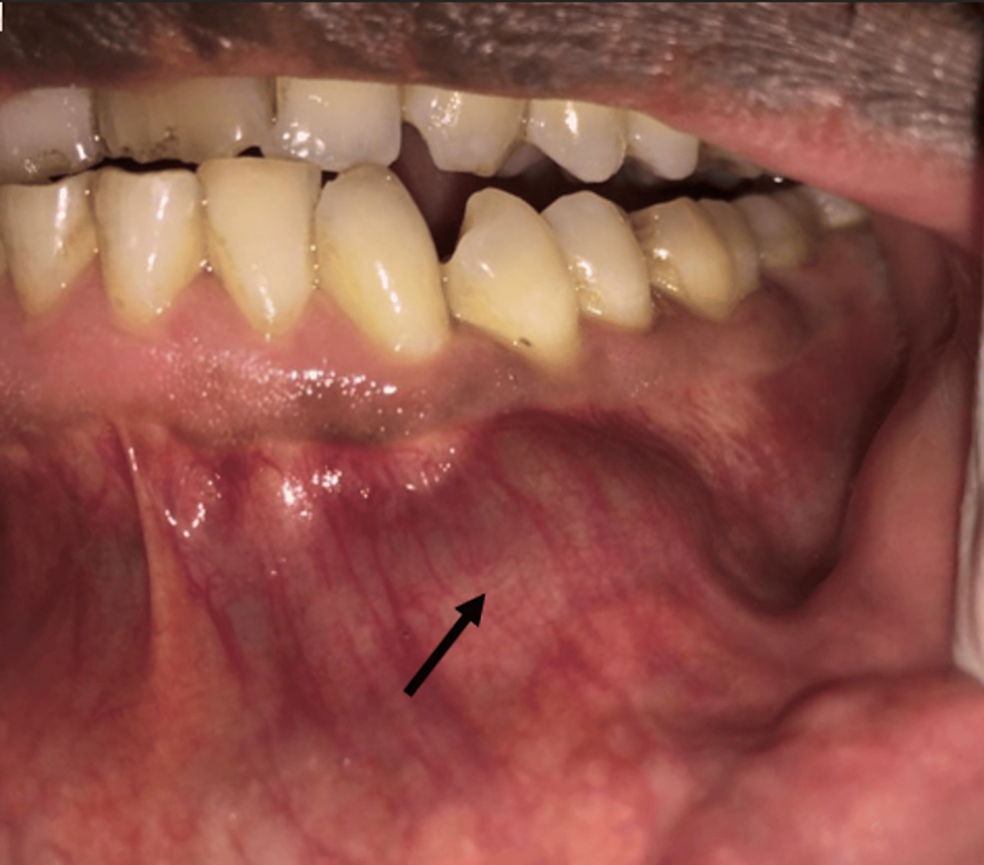
Non-tender swelling was noted over the lower left buccal aspect between 33 and 34 (FDI) FDI: Fédération Dentaire Internationale

**Figure 2:**
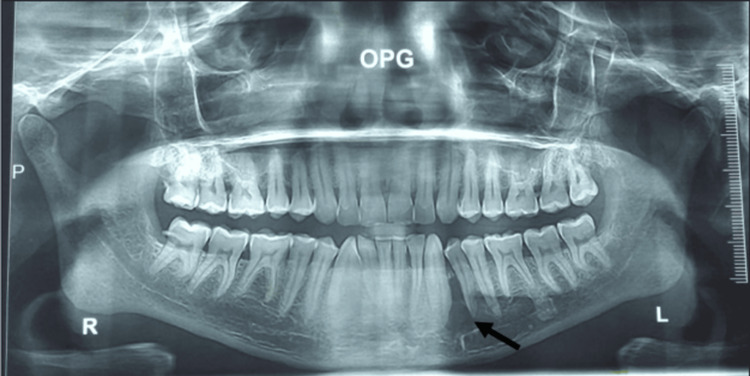
OPG depicted a well-defined, unilocular, completely radiolucent lesion with corticated margins presenting between 33 and 34 involving the periapical area OPG: orthopantomogram

A 39-year-old male with no co-morbidities presented to the outpatient department of oral and maxillofacial surgery with intraoral painless swelling that was noticed to be progressively increasing in size for two months. On extraoral examination, there was an absence of a fistulous tract with no signs of facial asymmetry or nerve paresthesia. Intraoral inspection revealed a solitary, firm, non-tender, oval swelling in the lower left side of the mandibular para-symphysis region concerning 33, 34, and 35 (Fédération Dentaire Internationale) teeth expanding buccally and measuring approximately 2.5x2 cm (Figure 1). The overlying gingiva and attached mucosa had no signs of inflammation, no draining sinus tract, and no ulcerations, and the mucogingival junction was not obliterated. There were no similar findings elsewhere in the oral cavity. Dental examination revealed intact, healthy enamel surfaces and grade I mobility with 33 and 34. Non-vitality was noted with an electric pulp tester for 34. Well-maintained oral hygiene with no stains or calculus. Radiology with an orthopantomogram depicted a well-defined, unilocular, completely radiolucent lesion with corticated margins present between 33 and 34 involving the periapical area (Figure 2). Routine blood investigations were within normal limits. Taking everything into consideration, the patient was counselled in the local language about the problem, and well-informed written consent was obtained. An incisional biopsy of the affected region was done. Histopathological examination determined the presence of multiple giant cells in clusters, suggesting a giant cell lesion.

**Figure 3:**
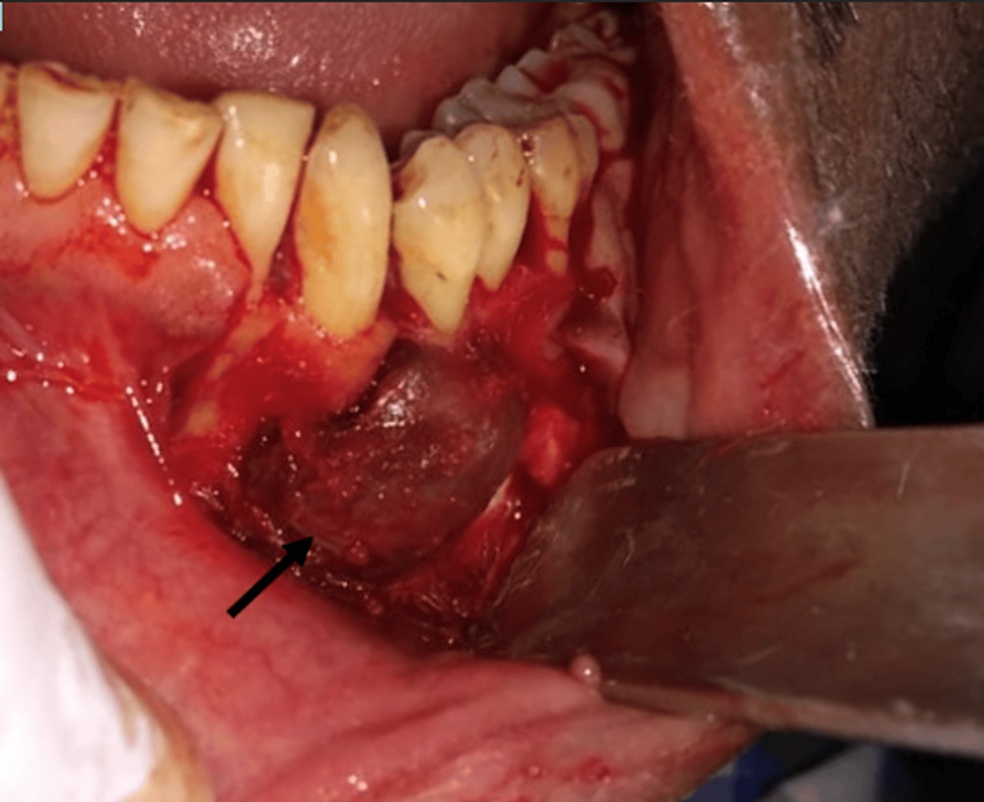
Full-thickness mucoperiosteal flap is elevated, exposing the lesion seen between 33 and 34

**Figure 4:**
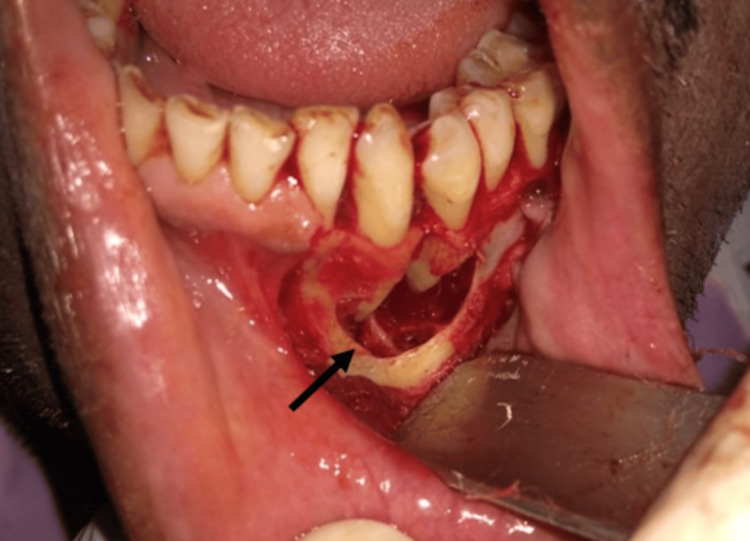
Surgical curettage was done; buccal bone defect noted between 33 and 34

**Figure 5:**
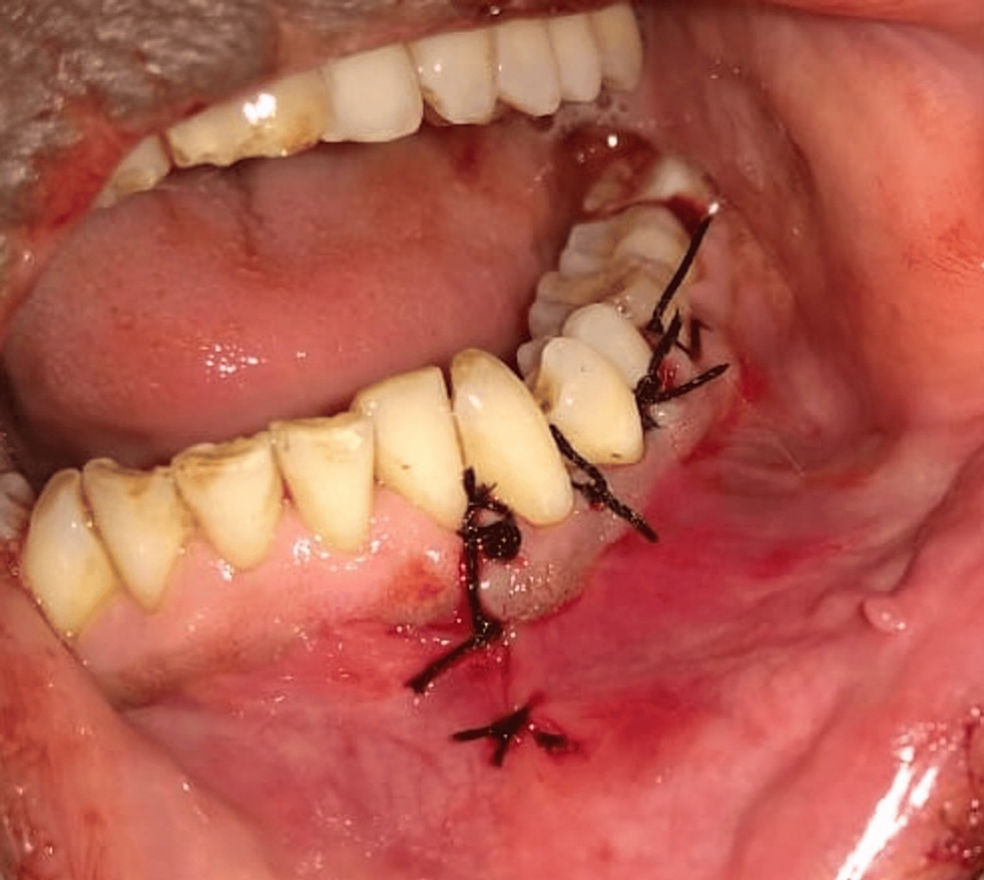
Primary closure of the flap done using non-resorbable round body 3-0 Mersilk interrupted sutures

**Figure 6:**
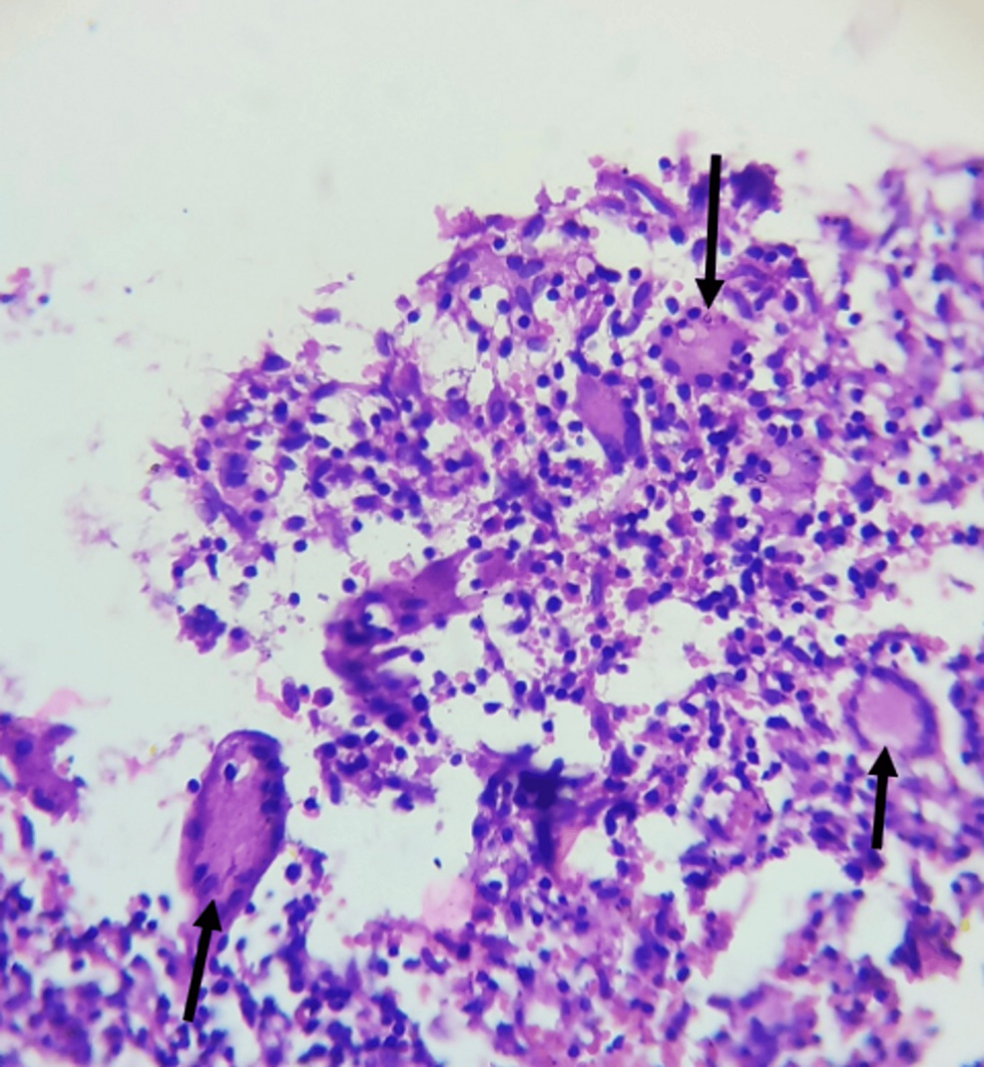
Histopathology determined the presence of multiple giant cells in clusters unevenly distributed in the background of spindle-shaped mesenchymal cells suggestive of CGCG CGCG: central giant cell granuloma

Considering the clinical, radiographic, and histological picture of the lesion, surgical curettage was planned under local anaesthesia. Following all universal aseptic precautions and antibiotic prophylaxis (amoxicillin 500 mg), a full-thickness mucoperiosteal flap was raised from 32 to 35 (Figure 3). Complete curettage of the lesion was carried out, preserving mental nerves and keeping 33 and 34 intact in their positions (Figure 4). Irrigation was done with saline and betadine at 10% w/v. Closure was done using non-resorbable 3-0 round-body braided Mersilk interrupted sutures (Figure 5). Follow-up was done after seven days; wound healing was satisfactory; thus, sutures were removed. Endodontic treatment was done with 33 and 34. The final histopathological findings were in support of a CGCG with multiple giant cells in a cluster unevenly distributed and separated by a spindle-shaped fibroblastic stroma (Figure 6). Thus, the patient was kept under regular follow-up for one year. No recurrence was noted during this period.

## Discussion

Literature indicates a higher incidence of misidentified lesions that resemble apical periodontitis, underscoring the importance of accurately diagnosing these conditions. The motive of this particular case report is to emphasize the significance of a clinician's expertise in differentiating non-endodontic lesions in this context to determine an appropriate treatment plan with a favorable prognosis.

CGCG is a rare, intraosseous, osteolytic lesion of the jaw. It has a wide range of ages in its manifestation; the majority of cases occur below the age of 30 years. This lesion has twice the female predilection over the male gender. Nearly 80% of lesions are present more commonly in the lower jaw anterior to the first premolars and rarely occur in the posterior segment of the jaws [1,12]. CGCG can also be localized to the jaws' tooth-bearing areas. In general, it is asymptomatic in the beginning stages but later can be expansile. CGCG is further classified based on clinical and radiological features as aggressive and non-aggressive CGCG. Aggressive CGCG exists when there is recurrence after enucleation and curettage with a lesion larger than 5.0 cm or if it includes a minimum of three of the following: pain, rapid growth, resorption of the root, displacement of teeth, perforation of the cortical bone, and recurrence. On the contrary, non-aggressive CGCG exists when the size of the lesion is smaller than 5 cm with the absence of symptoms and recurrences [13].

Radiologically, periapical X-rays display well-defined radiolucent, sharply demarcated, or sclerotic margins, showing their expansile nature sometimes between displaced tooth roots. Giant cell tumours are differentiated from CGCG by the presence of ill-defined calcifications within the lesion, denoting irregularly mineralized trabeculae. Multiple locules are appreciated in larger lesions [1].

CGCG consists of fibroblasts as primary tumour cells, which make up the proliferative component of the lesion. Multinucleated giant cells are in the background, while endothelial cells, dendrocytes, and macrophages are other miscellaneous cells in the histologic view. CGCG is histologically differentiated with an uneven distribution of multinucleated giant cells, forming clusters separated by spindle-shaped myofibroblastic stromal tissue. CGCG shows myofibroblastic differentiation in the stroma, which supports the behavior of the lesion as benign and thus can be included as a myofibroblastoma.

The case presented above was probably misdiagnosed as a periapical inflammatory lesion and thus referred for further management as the patient had painless swelling with normal tooth structure involved periapical in the mandibular anterior region. Such non-endodontic-origin lesions tend to grow more inciso-cervical than mesiodistally. Therefore, a clinician’s differential diagnosis should always include odontogenic keratocyst, dentigerous cyst, brown’s tumour of hyperparathyroidism, non-ossifying fibroma [14], giant cell tumour, and other lesions with giant cells histologically like cherubism, Paget’s disease, etc.

The choice of treatment for managing a CGCG has been surgical curettage until now. Surgical curetage usually involves damage to the jaw and teeth, which remains unavoidable, raising the chances of recurrence. Similarly, aggressive CGCG requires resection for a favourable prognosis. Thus, there is a requirement for alternate therapies to reduce the morbidity of patients in their older age groups, such as intravenous injections of corticosteroids, calcitonin, or interferon-alpha mentioned in the literature, which have variable success rates [15]. It has been reported that the number of giant cells, size of lesions, and osteoclastic hyperactivity are reduced along with the promotion of lamellar bone formation. Still, randomised clinical trials are very rare, with studies based on alternate therapies [11]. The case presented herein was an asymptomatic non-aggressive CGCG with a size less than 5 cm, intact lingual cortex and breached buccal cortex, and no displacement of associated teeth; thus, surgical curettage was considered as its management.

## Conclusions

CGCG is an uncertain, idiopathic, destructive lesion of the jaws. Although we are making significant progress in improving our knowledge of this giant cell lesion, there is still much to discover about its pathophysiology. Surgical curettage is still the most common therapy followed in cases of non-aggressive CGCG and resection in aggressive CGCG, despite undesirable damage to the jaw or teeth. However, non-surgical therapeutic alternative trials, especially inhibitors of osteoclastic activity, have to be worked out with authenticity. It is essential to make an accurate diagnosis and exclude lesions that present in a similar manner.
